# Ancient genomic architecture for mammalian olfactory receptor clusters

**DOI:** 10.1186/gb-2006-7-10-r88

**Published:** 2006-10-01

**Authors:** Ronny Aloni, Tsviya Olender, Doron Lancet

**Affiliations:** 1 Department of Molecular Genetics and the Crown Human Genome Center, The Weizmann Institute of Science, Rehovot 76100, Israel

## Abstract

**Background:**

Mammalian olfactory receptor (OR) genes reside in numerous genomic clusters of up to several dozen genes. Whole-genome sequence alignment nets of five mammals allow their comprehensive comparison, aimed at reconstructing the ancestral olfactory subgenome.

**Results:**

We developed a new and general tool for genome-wide definition of genomic gene clusters conserved in multiple species. Syntenic orthologs, defined as gene pairs showing conservation of both genomic location and coding sequence, were subjected to a graph theory algorithm for discovering CLICs (clusters in conservation). When applied to ORs in five mammals, including the marsupial opossum, more than 90% of the OR genes were found within a framework of 48 multi-species CLICs, invoking a general conservation of gene order and composition. A detailed analysis of individual CLICs revealed multiple differences among species, interpretable through species-specific genomic rearrangements and reflecting complex mammalian evolutionary dynamics. One significant instance involves CLIC #1, which lacks a human member, implying the human-specific deletion of an OR cluster, whose mouse counterpart has been tentatively associated with isovaleric acid odorant detection.

**Conclusion:**

The identified multi-species CLICs demonstrate that most of the mammalian OR clusters have a common ancestry, preceding the split between marsupials and placental mammals. However, only two of these CLICs were capable of incorporating chicken OR genes, parsimoniously implying that all other CLICs emerged subsequent to the avian-mammalian divergence.

## Abstract 2

A new tool for genome-wide definition of genomic gene clusters conserved in multiple species was applied to olfactory receptors in five mammals, demonstrating that most mammalian olfactory receptor clusters have a common ancestry.

## Background

Olfactory receptor (OR) genes constitute the largest superfamily in the vertebrate genome, with several hundred genes per species [1-3]. This large repertoire of receptors mediates the sense of smell through the recognition of diverse volatile molecules, used to detect food, predators, and mates. Mammalian OR genes reside in about 50 genomic clusters of one to several dozen genes, which are dispersed among many chromosomes [4,5]. Although the number of clusters is similar among species, the typical cluster size varies significantly because of extensive lineage-specific evolutionary events (for example, inter- and intra-chromosomal gene duplications and genomic deletions) [3,6-8].

Comparative analysis of mammalian OR clusters is crucial for deciphering the common evolutionary origins of the OR repertoires, as well as for highlighting inter-species differences.

Large-scale comparisons have mapped most pairwise relations among human and mouse clusters based on sequence similarity between individual genes [9]. A similar study also revealed that, in most cases, pairs of OR clusters that exhibit human-mouse similarity fall into established synteny blocks, which indicates their common origin [10]. Clusters with similarity that did not share synteny relationship were attributed to inter-chromosomal duplication events. Similarly, the combination of synteny data and sequence similarity has been used to map between the majority of human and dog clusters, indicating their common origin [11]. Thirteen dog clusters that could not be mapped were suggested to be 'dog specific'.

A highly relevant endeavor is the recent establishment of a comprehensive network of whole-genome pairwise alignment chains, bridging between local sequence similarity and global synteny mapping, thus providing a better resolution for genome-wide comparisons [12]. Because this system currently includes all complete mammalian genomes published so far, including the marsupial opossum (*Monodelphis domestica*), it has the potential to assist greatly in conducting a comprehensive multi-species comparison of mammalian OR clusters. Here, we used this powerful framework to establish relationships among mammalian OR clusters on a genome-wide basis. This allowed us to reconstruct a parsimonious scenario for the evolution of gene clusters in the mammalian olfactory subgenome, and to reconstruct a putative OR cluster architecture of the common ancestor of five mammals, spanning nearly 200 million years of phylogeny.

## Results

### OR genomic mining in opossum and dog

For the OR gene repertoire of the opossum *Monodelphis domestica*, we mined a total of 1,518 ORs (the nucleotide and protein sequences are available in Additional data files 9 and 10) from the Opossum October 2004 assembly (monDom1). This was achieved using previous computational methodologies, as described previously [3,13]. Because the opossum genome has not been assembled to the chromosome level, the sequence coordinates were referred to genomic scaffolds. The assembly used consisted of scaffolds with average length of about 4.5 megabases (Mb), ensuring inclusion of whole OR clusters or substantial parts thereof in most cases.

Our previously reported canine OR repertoire [14] was a result of combining directed DNA sequencing of the beagle genome and data mining of Celera's 1× poodle genome, and it contained 997 ORs sequences without genomic location. For the purposes of the present study, we re-established the repertoire from the July 2004 assembly of the boxer breed (canFam1). We applied BLAT (BLAST [Basic Local Alignment Search Tool]-Like Alignment Tool) and other procedures as described previously [13], using the published canine ORs as queries. The new dataset obtained included 922 ORs (the nucleotide and protein sequences are available in Additional data files 11 and 12). The two repertoires were compared using Sequencher (version 4.2 for PC; GeneCodes Corp., Ann Arbor, Michigan, USA) with a 97% identity threshold to yield an overlap set of 765 ORs. The main reason why 189 of the poodle ORs failed to overlap the boxer genome is low sequence quality, mainly at the ends of the unmatched poodle ORs. The 209 ORs found in the new mining effort were classified into families and subfamilies and were assigned an appropriate symbol, using the nomenclature system of HORDE (Human Olfactory Receptor Data Exploratorium) [13]. The opossum and dog OR sequences are available in the HORDE database [15] and in Additional data files 9, 10, 11, 12.

### Identification of clusters in conservation

We aimed to produce a systematic depiction of the relationships among OR clusters of five mammalian species. For that we developed a three-step algorithm to identify CLICs (CLusters In Conservation), the multi-species equivalent of a genomic cluster. This algorithm progressed from the intra-species identification of genomic clusters, through the pairwise comparison of individual ORs from different species, to integration in the multi-species framework of CLICs.

In the first step, we defined OR clusters in all five species, based on a selected maximal intergenic distance of 300 kilobases (kb). This resulted in the definition of 48 ± 5 (mean ± standard deviation) clusters with two or more ORs and 24 ± 9 singletons in the four placental mammals (Table 1). For opossum, the numbers were considerably greater, presumably because the fragmented genome assembly in this species (Table 1).

**Figure 1:**
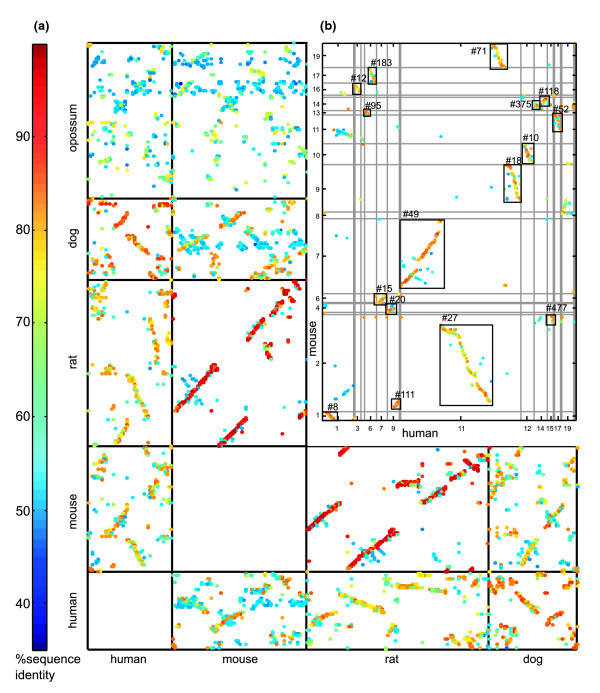
Conservation of synteny of OR genes. **(a) **All ORs from each species are ordered along the axis according to their genomic location from chromosome 1 to X (or by scaffold number in the case of the opossum), and by the internal megabase coordinates in each chromosome. Each point represents an alignment between two ORs from different species in the UCSC alignment net, colored according to the degree of DNA sequence identity (x-axis for the reference species, y-axis for the target species). Diagonals in both directions represent conservation of gene order, whereas reverse diagonals indicate a reverse of gene order relative to the 'plus' DNA strand. Off-diagonal points generally indicate micro-rearrangements, but those that are associated with low percentage identity possibly represent alignment errors. **(b) **Zoomed human versus mouse comparison, with chain numbers (by UCSC hg17 versus mm6 alignment net) indicated for the 16 alignment chains that contain at least six pairs of syntenic orthologs. Chains #95 and #183 represent disrupted synteny, because the alignment of a succession of ORs from human chromosome 6 is split between mouse chromosomes 13 and 17 (as described by Amadou and coworkers [26]). Chains #375 and #118 capture a genomic inversion. OR, olfactory receptor.

The second step was focused on relationships stemming from the UCSC (University of California at Santa Cruz) alignment net for 12 species pairs [12]. This net is a whole-genome pairwise alignment protocol that provides the best match to every position in the genome, according to both local sequence similarity and global genomic context. Of 5,969 ORs in five species, 5,305 (89%) were found to match an OR in an alignment net with at least one other species (Table 2). A small fraction (3.5%) of alignment pair events were between an OR and a genomic sequence not hitherto defined as an OR gene (see the legend to Table 2). The aligned ORs are shown in Figure 1 in a genomic position context, in which each panel shows a whole genome comparison of two species. The visible contiguous diagonal arrays of OR genes, often spanning considerable genomic segments, provide evidence for the conservation and syntenic organization of OR clusters in different mammals. Synteny often extends beyond the OR clusters, whereby the relevant alignment chain contained non-OR genes as well. For example, this was found to be true by manual examination for 30 out of all 33 human versus mouse chains.

The inter-species OR alignment pairs were filtered to highlight ORs with high confidence of orthology, defined here as 'syntenic orthologs', which correspond to well defined synteny blocks in addition to high mutual sequence identity. The final subset of syntenic orthologs contained OR pairs that belong to alignment chains longer than 100 kb and showing sequence identity higher than a 72% cutoff. Approximately 56% of all ORs (and 71% of the eutherian ORs) were included in the syntenic orthologs category.

Finally, in the third step, CLICs were defined as connected components in an OR graph. A CLIC is thus a set that includes all OR clusters from different genomes, within which every cluster is connected by at least one syntenic orthology edge to at least one other cluster. Whenever several genes from the same species were aligned to a single gene in another species, and were defined as its syntenic orthologs, they were all included in the same CLIC.

**Figure 2:**
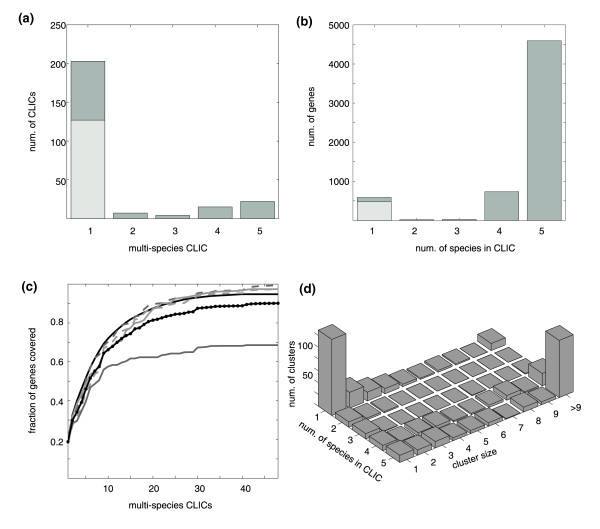
CLIC statistics. **(a) **Different types of CLICs are characterized by the number of species involved. The fraction of opossum-specific CLICs is indicated by light gray. **(b) **The total number of genes in CLICs from each type. The opossum-specific fraction is indicated as in panel a. **(c) **Cumulative plots show the fraction of OR genes that is covered by multi-species CLICs of decreasing size (sorted first according to the number of genes in human, and then by the numbers in mouse, rat, dog, and finally opossum). All multi-species CLICs together cover more than 95% of any eutherian OR repertoire (solid black = human, dashed dark gray = mouse, dashed light gray = rat, solid light gray = dog), but only two-thirds of the opossum repertoire (solid dark gray). The coverage of the combined repertoire of all species is shown by black circles. **(d) **The total number of clusters included in CLICs from each type and size. CLIC, clusters in conservation.

The foregoing analysis divided the examined mammalian OR repertoire into 251 mutually exclusive CLICs (Figure 2a,b, and Additional data file 1, with sample data in Table 3). Of these, 48 CLICs contained clusters from more than one species (multi-species CLICs), with most of them containing representations from all five mammals, or at least the four placental mammals. The multi-species CLICs encompassed 90% of the combined mammalian OR repertoire (Figure 2c). These results suggest a significant overall mammalian conservation of the cluster configurations, and lead to the inference that many of the OR clusters were present in the evolutionary common mammalian ancestor(s). As a caveat, we note that our analyses, based on large-scale genome alignments, are sensitive to cases of incompleteness of genome assembly.

A single species CLIC may represent a cluster that was not present in the inferred common ancestor, but was introduced more recently into a particular lineage. Although larger genomic clusters were usually assigned to multi-species CLICs, singleton ORs and small clusters often appeared as single species CLICs (Figure 2d).

**Figure 3:**
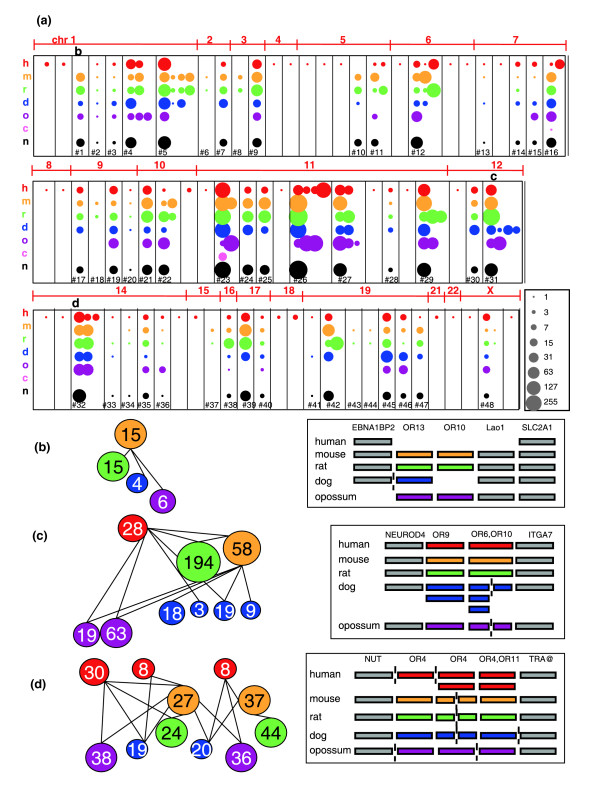
CLICs of OR genes. **(a) **CLIC (columns) are shown by human genomic order (see Table 3), with human chromosome numbers indicated (top ticked line). For CLICs that do not contain human clusters, the order was determined by the human location that is syntenic to the region of the mouse OR cluster (Additional data file 1). For each species (h = human, m = mouse, r = rat, d = dog, o = opossum, c = chicken, n = consensus gene count) circle size is proportional to log2(n - 1), where n is the number of genes in the OR clusters within the CLIC. All multi-species CLICs are enumerated (#i at bottom); nonhuman single species CLICs are not shown. **(b-d) **Detailed depiction of three CLICs indicated by the corresponding capital letter above the CLIC column in panel a. To the left of panels b-d, clusters are represented by circles (colored for species, as in panel a), with gene count indicated. Lines connect every two clusters sharing syntenic orthologs. To the right of panels b-d are schematic genomic representations of the clusters, with OR gene groups in species color and OR family indicated. Grey bars represent flanking non-OR genes (HUGO nomenclature symbols indicated [57]); TRA@ is the T-cell receptor alpha locus. Multiple rows for the same species indicate the inclusion of clusters from multiple chromosomes in the CLIC. A break in local or large-scale synteny is marked by a broken line. For the complete list of the genomic coordinates of all analyzed genes, see Additional data file 2. CLIC, clusters in conservation; OR, olfactory receptor.

The number of genes from each species in a given CLIC varied considerably (Figure 3). Attempting to obtain an overview on cluster sizes in the different species, we preformed an analysis that focused on larger CLICs. This was done to filter noise stemming from small number statistics. Considering CLICs with at least 15 human genes (containing 80% of all genes in multispecies CLICs), human and dog had a similar gene number in a given CLIC, whereas mouse and rat had a larger number (typically 1.5-fold higher). Thus, the observed inter-species variation in repertoire size (Table 1) cannot be explained by the number of clusters but rather by increased cluster size. This is in accordance with previous results [10,16].

### Analysis of evolutionary events within CLICs

The definition of CLICs generates a common framework, within which species-specific evolution of OR clusters can be analyzed (Figure 3). A close examination of the CLICs reveals events such as cluster duplication, cluster deletion, and cluster splitting. The relevant evolutionary scenarios include unitary events (for instance, a genomic deletion in a single lineage) as well as complex events that occurred along more than one lineage. Nevertheless, absence of a CLIC from a genome may result from an assembly problem; this is particularly relevant to the opossum genome.

Cluster deletion is evident for CLIC #1, which contains one conserved OR cluster in all mammals except human (Figure 3b). A human-specific cluster deletion appears to be the best explanation, because otherwise there is a clear synteny relationship in this region for all five species examined (Figure 3b). We performed a BLAST search of the mouse OR protein sequences of this CLIC against the human repertoire, but the matches were of low sequence similarity (around 50% identity), supporting the absence of any human orthologs. This human-specific deletion of an OR cluster is intriguing because in mouse the relevant OR cluster on chromosome 4 was tentatively associated with the capacity to smell isovaleric acid [17,18], an odorant that many (but not all) humans can detect [19].

Inter-chromosomal cluster dispersion is observed for CLIC #31 (Figure 3c). It contains one OR cluster from every species except dog, whereas dog is represented by four clusters. Two of the dog clusters belong to two different human-dog synteny blocks, with the breakpoint located at the middle of the human OR cluster. For the two other clusters there is no conserved synteny beyond the stretch of OR genes. These inferred novel OR locations in the dog genome could be created by an inter-chromosomal cluster duplication, or by movement of part of the cluster. In addition, four dog-specific CLICs (#113, #115, #116, and #123; see Additional data file 1) with a similar subfamily composition (belonging to the OR6 and/or OR9 families) might also have been created by a partial cluster duplication originating in CLIC #31. However, these CLICs belonged to short local alignments, and therefore were not integrated into CLIC #31. Family OR6 has greatly expanded in the rat lineage too, in this case within a single cluster assigned to CLIC #31 (Figure 3c).

Another example of cluster duplication is CLIC #32, which contains two clusters from each of the nonhuman species, whereas in human there are three clusters, two of which (chr14@19.5, chr15@19.8) are highly similar to each other (Figure 3d). This CLIC appears to capture a recent event of cluster duplication in the human lineage, as previously suggested, based on a similarity in the subfamily content [3]. Indeed, all members of the two human clusters showed at least 90% mutual protein identity, which is a very high score. In parallel, the best mouse hits for most members of the two human clusters were found in a single mouse cluster (chr14@45.4). These results further support evidence of cluster duplication in human lineage.

In addition, genes from family OR4 are divided in a different way between the two clusters of each species, although they still belong to one CLIC (Figure 3d). This is consistent with the notion that the two clusters were originally on the same ancestral chromosome, as is indeed the case for human chromosomes 14 and 15 [20]. Chromosomal translocation was suggested to be a possible mechanism for fragmentation of a single genomic cluster into smaller clusters, whose ORs are from a common phylogenetic subfamily [21].

### The reconstruction of the ancestral olfactory subgenome

For the purpose of reconstructing the probable ancestral olfactory mammalian subgenome, we considered all multi-species CLICs excluding six that appeared only in the two closely related rodents (Additional data file 1). These 42 CLICs were inferred to be present in the eutherian common ancestor genome. However, we cannot rule out the possibility that a single species CLIC existed in the ancestral genome but was lost in all but one species. Such hypothesis may be especially valid for the dog-specific CLICs, for which only one event of cluster deletion in the human and rodents lineage is required, after the split from the dog. We therefore conducted a BLAST search with the 20 protein sequences of the 12 dog-specific CLICs against the human, mouse, and dog OR repertoires. Ten of these ORs are probably recent duplications in the dog OR repertoire, exhibiting high protein identity (>90%) to other dog ORs. The other ten genes were in general closer to their dog hit in comparison with human and mouse.

Among the 42 multispecies CLICs, 26 were common also with opossum and were inferred to represent ancestral clusters in the last common ancestor of eutherians and marsupials. Less than one quarter of the opossum OR clusters (36 out of 163) were integrated into multispecies CLICs, as compared with 74% of all eutherian clusters (212 out of 288). In order to examine the likelihood of an ancestral origin of the remaining opossum clusters, we examined the opossum clusters disregarding the previously employed CLIC definition constraints. Most of the opossum-specific CLICs (96 out of 127) were not found at all on the opossum-human or opossum-mouse alignment nets. These CLICs contained 232 ORs (out of a total 1,518 ORs in opossum), and ranged in size from 1 to 37 genes (Additional data file 1). At least 54 ORs of this group belonged to a unique expansion in the opossum genome, which exhibited low sequence similarity to eutherian genes (an average of 48% identity at the protein level). The other ORs belonged to OR subfamilies shared with eutherians, which were probably excluded from the alignment net because they were too divergent at the DNA level or because of assembly artifacts. Indeed, two-thirds of these scaffolds were less than 100 kb long. We found that 91% of the entire opossum genome is included in human-opossum alignment chains larger than 100 kb [22]. This is in good agreement with our finding that 1,340 out of 1,518 ORs (88.2%) are included in multi-species CLICs.

Each of the 31 remaining opossum-specific CLICs was merged with a predefined multi-species CLIC, which contained the gene with the highest sequence similarity in the human-mouse alignment net. No minimum sequence identity or chain length was required. As a result, the additional opossum clusters joined 20 multispecies CLICs; 13 of the target CLICs were devoid of opossum cluster beforehand (dAdditional data file 1). Although this procedure may lead to the inclusion of false positives, the finding still provides evidence suggesting an early mammalian origin of 38 out of the 42 inferred ancestral clusters, and suggests that four CLICs (#14, #17, #39, and #42) are eutherian specific. However, the latter conclusion should be taken with caution, given the incomplete disposition of the opossum genome assembly.

**Figure 4:**
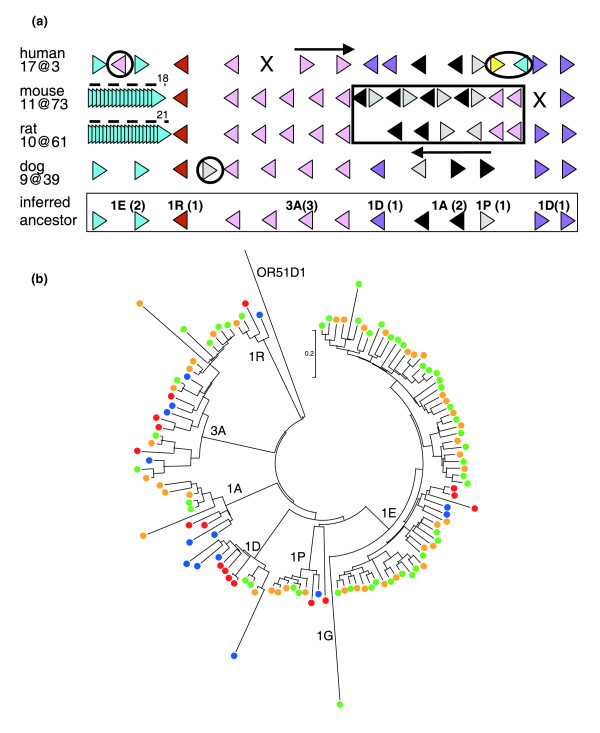
The reconstruction of an ancestral cluster. **(a) **Detailed comparison of gene content and order is shown for the four clusters included in CLIC #39 (see Table 3), containing genes from the OR1 and OR3 families. A schematic ancestral configuration is shown at the bottom row, with inferred minimal number of genes. Genes are presented as triangles colored by subfamily affiliation (bottom row; yellow for subfamily OR1G). Inferred chromosomal rearrangements relative to the ancestor are specified for each species: circle = insertion, X = deletion, arrow = inversion, broken line with number of genes = tandem duplication. A complex duplication in rodent genomes, involving subfamilies OR3A, OR1P, and OR1A, is marked with a rectangle. This duplication was probably formed via several events, some of which occurred after the split between mouse and rat lineages. The same region had experienced another independent event in the dog genome, in which three genes from subfamilies 1A and 1P were inverted as one unit. Tandem duplication in one end of the rodent clusters forms a series of numerous adjacent highly similar genes from the same subfamily (OR1J). The human and mouse orthologous clusters were studied and compared previously [27], and a complex orthology relationship among the genes was described. **(b) **A phylogenetic tree of CLIC #39 ORs from which the ancestral cluster gene count can be inferred. The phylogenetic tree was generated with Mega version 3.1 [58] using ME algorithm, and Poisson correction for distance calculation. Protein sequences were aligned with Clustalx [59]. The colors of circles next to the phylogenetic branches indicate species (blue = dog, green = rat, orange = mouse, red = human). OR51D1 serves as an out-group. CLIC, clusters in conservation; OR, olfactory receptor.

For each of the 42 inferred ancestral clusters, an ancestral gene count was estimated, using a simple statistic derived from the cluster size distribution of the corresponding CLIC (Table 3). We note that assessing the number of genes in ancestral clusters is problematic, because contemporary clusters reflect an ongoing process of gene duplication and deletion, not necessarily at the same rate. With this caveat, it appears that the mammalian ancestor had approximately 1070 OR genes. Of these, 38% were disposed in two large clusters of more than 100 genes (CLIC #23 and CLIC #26), 59% in medium size clusters of 7-44 genes, and the remaining 3% being in small clusters of one to six genes. It is also possible, with appropriate caution, to reconstruct the internal organization of the ancestral clusters (Figure 4 and Additional data file 4). Such reconstruction indicates signatures of lineage-specific genomic reorganization, including tandem duplication of individual OR genes, inversions, insertions, and deletions.

### Chicken-mammal conservation

The chicken OR repertoire was found to contain 554 genes, of which 476 (86%) were pseudogenized and only 78 had intact open reading frames [7,23]. The chicken OR repertoire was highly restricted, with 75% of the genes belonging to a single family (a newly defined family OR14; Olender T and coworkers, unpublished data). Only 8% of the chicken ORs were assigned a genomic location, even though 90% of the total chicken genomic sequence was contained within assembled chromosomes [7]. The failure of the majority of the chicken ORs to undergo whole-genome shotgun assembly probably stems from their high mutual sequence similarity.

The CLIC-defining algorithm was applied to the chicken OR gene repertoire. The cutoff of chain length was lowered to 50 kb, and no sequence similarity cutoff was used beyond the maximal expectation value embedded in the alignment chain definition. Only two chicken clusters (with a total of 13 OR genes) could be joined to the previously defined mammalian CLICs (Figure 3a and Additional data file 5). Most of the remaining chicken ORs, including those missing a genomic location, could not be aligned beyond the OR coding region. Half of them were included in chains of 1,000-50,000 base pairs (bp) long, and hence they had the potential to contain an entire 1 kb OR coding region (Additional data file 6). This finding is perhaps unsurprising, given that most of the chicken ORs belong to chicken-specific expansion.

The largest chicken cluster, with 12 class I ORs (including four pseudogenes), belonged to CLIC #23 (Additional data file 5), and was included in an alignment chain that spanned 285 kb on chicken chromosome 1 and 2,500 kb on human chromosome 11 (with 103 human ORs). This chain also contained the syntenic β-globin cluster, with four chicken β-globins as compared with five human genes [24,25]. The second match between chicken and mammalian clusters was in CLIC #16, which contained a single OR from chicken chromosome 1 (belonging to subfamily OR10AC) aligned to human OR10AC1P on chromosome 7 (Additional data file 5). The human genomic region, related to the relevant alignment chain, contained six human OR genes (included in CLIC #16) and five bitter taste receptor genes. Of these, only one OR (OR1AC1P) and one taste receptor (TAS2R49) appeared in the human-chicken alignment net, indicating their conserved synteny. In addition, this chain included two conserved ephrin receptors (EPHB6 and EPHA1).

## Discussion

The identification of orthology relationships among OR genes has been recognized previously as a complicated task [6,26,27]. OR orthologs have been defined for several pairs of genomes on the basis of amino acid sequence similarity [4,8,10,28]. However, signals of high sequence similarity among true orthologs are obscured in this large gene superfamily by extensive gene duplication as well as gene conversion and sequence divergence. A recent multi-species approach for ortholog identification increased the robustness of inference, by seeking three-way dog-human-mouse mutual best hits [14]. Naturally, such a strict requirement also reduced the sensitivity of detection. Alternative algorithms for large-scale orthology identification, such as COG [29], INPARANOID [30], and OrthoMCL [31], entailed complex many-to-many orthology relationships within a group of proteins but also relied solely on mutual coding sequence similarity. Enrichment by gene-related structural or functional data has proven effective in orthology determination [32,33], but it is impractical in the case of the OR genes because of the paucity of relevant information.

In the present study we took a novel approach that introduced the use of global synteny on top of local sequence similarity. Based on whole-genome pairwise alignments among five mammals, pairs of syntenic orthologs were identified with high confidence, supported by the conservation of genomic location. Applying the connected component algorithm to syntenic ortholog pairs from all species captured the intricate relationships within the OR gene superfamily, as manifested in the definition of CLICs. This resulted is groups of ORs presumably derived from a specific genomic location in a presumed evolutionary ancestor. We note that our conclusions are based on the assumption that very limited interaction/swapping of sequences has occurred among genes and clusters, for instance by gene conversion.

Another concept that we adopted to deal with the complexity of the OR gene superfamily is the definition of an evolutionary common ancestor at the cluster level rather than at the gene level. Common ancestry of similar clusters has previously been inferred only with regard to pairs of species - human versus mouse [9,10] or human versus dog [11] - or to specific clusters [34,35]. It has also been observed that the number clusters is surprisingly similar among mammals, despite considerable variation in the total repertoire size [4]. An important advance presented here is the definition of multi-species sets of conserved clusters, providing one-to-one mapping among clusters of different species. These newly defined CLICs revealed evidence of an ancestral evolutionary origin of the mammalian OR clusters, rather than independent cluster formation in each lineage. It suggests that the uniform number of mammalian clusters stems from an ancestral common architecture that remained practically unchanged in contemporary species.

The CLIC framework was found to apply also to the OR repertoire of the more ancient opossum. Hence, the formation of the OR cluster architecture appears to have taken place before the split between marsupials and eutherians 185 million years ago. Importantly, the analysis at the cluster level revealed a conservation signal that could hardly be detected at the individual gene level, because of the relatively high (approximately 40%) DNA sequence divergence in human-opossum pairs of OR coding regions (Additional data file 7). However, in contrast to other species, ORs in the opossum formed numerous additional clusters that could not be assigned to the shared set of CLICs. This phenomenon could represent lineage-specific expansion of the marsupial repertoire or, alternatively, loss of ancestral clusters from the eutherian lineage. Finding out which of these alternative scenarios is correct could be aided by an outgroup genome such as that of the monotreme platypus *Ornithorhynchus anatinus *[36]. We note that current fragmentation of the opossum genome assembly could be an alternative reason for hampering proper CLIC joining of opossum ORs.

The question of a potential origin of OR clusters beyond the mammalian lineage has been addressed here by broadening the comparative analysis to the chicken OR repertoire. Accordingly, only one nonsingleton cluster, which includes class I receptors, has an evident common origin with a corresponding mammalian cluster. This cluster was previously suggested to be the most ancient olfactory cluster [3]. The inability to identify CLIC relationships for other clusters in the chicken genome could be due either to considerable repertoire divergence after the mammalian-avian split or to massive OR gene loss in the avian lineage. The latter is supported by a relatively poor diversity and massive pseudogenization of the chicken OR repertoire [7,23]. We have also begun to analyze the OR repertoire of the frog *Xenopous tropicalis *[7], which currently is too fragmented to allow CLIC analysis. However, we were able to discern considerable diversity, with practically all human-defined OR gene families amply represented (unpublished data). This result, which is in agreement with previously published work [7], may indicate that a rich OR repertoire existed before the amphibian-reptilian split, providing further support to the chicken OR loss scenario.

The CLIC analysis provides a framework for a further level of analysis beyond evolutionary conservation, namely the study of variability among repertoires. The ongoing process of 'birth and death' of genes leads to large fluctuations in the number of functional receptors [37]. As the diversity of the OR repertoire may serve as an indication for functional olfactory acuity of an organism [4,38,39], comparing variability at the cluster level (for instance, rearrangements within clusters and loss or gain of complete clusters) would help to discern potential functional differences among species. An example reported here is the loss of a complete cluster from the human lineage. A presumed syntenic mouse genomic cluster belonging to CLIC #1 was associated with smelling isovaleric acid [17,18]. However, because humans are still capable of detecting this odorant, it is possible that OR(s) from another cluster compensates for this loss.

The increase of repertoire size can occur via two main processes: expansion within clusters, or dispersion to new genomic locations. The former appears to dominate the increase of the rodent repertoire, as illustrated by a consistent excess of rodent genes in mammalian CLICs. Extensive tandem gene duplication in rodents was pointed out previously as a dominant factor in OR evolution [8,10,16]. The present study further relates this process to the variation between mouse and rat repertoire sizes, which appears to have arisen mainly from a dramatic expansion of a single rat cluster (CLIC #31). This may represent an enhanced recognition or discrimination of the rat toward a specific set of odorants, potentially related to a species-specific ecologic/behavioral niche.

Cases of lineage-specific clusters have previously been described for the human repertoire [40,41]. A similar phenomenon has been demonstrated here by several dog-specific CLICs that represent an expansion of subfamily OR6C to eight distant locations in the dog genome. Interestingly, the same subfamily has been amplified independently via an inter-chromosomal process in the dog genome, and via an intra-chromosomal duplication within a single rat cluster.

We considered whether our analysis identifies evidence for a single OR that seeded the evolution of a cluster. Such a scenario might appear as a CLIC composed of a single gene in one lineage and more in others. We identified one case, namely CLIC #3, which matches the suggested scenario, with one OR in the mouse and two to four ORs in the other species. However, this situation is indistinguishable from a species-specific deletion.

An important finding of the present analysis is that OR clusters represent an ancient genomic architecture of the mammalian genome. This conserved feature implies biologic importance, potentially related to a common regulatory mechanism of gene expression control [42-45]. Further support for this notion derives from the observation that the primate-specific OR7E subfamily, composed chiefly of nonfunctional pseudogenes, shows a much sparser cluster architecture, with a considerable number of singletons. One mechanism of cluster generation and propagation is related to genomic sequence repeats [46]. It is noteworthy that shared clustering appears despite the diversity of repeat elements in different mammalian genomes [47,48].

The correct description of evolutionary relationships among mammalian OR clusters is important for an additional reason; it could provide a useful avenue to the identification of regulatory elements. The framework of CLICs provides a natural set of orthologous sequences for the identification of ANCORs (ancestral noncoding conserved regions [49]) within an individual OR cluster. Such elements are appropriate candidates for a regulatory role, such as transcription regulation or post-transcriptional modification. A great challenge in the study of ORs is to elucidate the regulatory mechanisms that mediate exclusive expression of a single allele of one receptor per olfactory neuron. Exploring ANCORs within CLICs may suggest putative key players in this process.

## Conclusion

The genomic architecture of mammalian OR gene clusters has an ancient evolutionary origin, preceding the marsupial-eutherian split. Species-specific evolution has further shaped the different olfactory subgenomes, both via gain and loss of complete clusters, and via expansion and contraction of existing clusters. The framework of CLICs enables one to pinpoint genomic commonalities and differences among species, and potentially relate them to olfactory capabilities. The same approach may also be applicable for other gene superfamilies.

## Materials and methods

### OR genes and clusters

#### Human

The complete human OR repertoire with 851 genes and pseudogenes, including genomic coordinates mapped onto the May 2004 (hg17) assembly, were extracted from the HORDE database [13]. Subfamily OR7E (86 genes), representing a primate-specific expansion [41], were eliminated from the analysis.

#### Mouse and rat

A total of 1,296 mouse ORs were kindly provided by Zhang and Firestein [18] (accession numbers AY072961-AY074256). A total of 17,58 rat ORs [16] were kindly provided by J Young and B Trask, University of Washington (accession numbers are detailed in Additional data file 8). We assigned their genomic locations on the mouse March 2005 assembly (mm6) and rat June 2003 assembly (rn3) using BLAT [50].

#### Chicken

A total of 554 chicken ORs were used as described [7], with their published coordinates on the chicken February 2004 assembly (galGal2). For the purposes of this study, genes that were mapped to an undefined chromosomal location (the virtual chromosomes 'chrUn' and chromosomes with the suffix 'random') were filtered out (Table 1).

For each of the six repertoires (Table 1), sets of OR genes located on the same chromosome with no more than 300 kb distance between consecutive genes were identified as OR clusters, including singleton clusters with a single gene. Because the number of identified clusters decreases as a function of the maximal intergenic distance allowed, we selected a distance criterion of 300 kb, at which the rate of this decrease becomes more moderate (Additional data file 3). The genomic coordinates of all analyzed ORs and their assignment to clusters are available in the HORDE database [15] and in Additional data file 8.

### Data mining procedures of opossum ORs

The first 500 ORs were identified based on the UCSC human-opossum net alignment net (assembly hg17 versus monDom1). A BLAT search [50] was performed using all 499 opossum ORs that were found to represent true OR sequences after translation into proteins. All hit locations were then extracted from the genome and subjected to further protein translation procedures.

The first TBLASTN search [51] was performed using the following 24 OR sequences: cOR4Z2, cOR9S6, MOR177-3, MOR220-1, MOR248-10, MOR263-4, MOR264-6, and an additional 17 consensus sequences representing the 17 human OR families. The second, third, and fourth included 30, 17, and 65 opossum ORs, respectively. The criterion for choosing a particular OR to serve as query was that it would represent the OR subfamily that was not included in the previous rounds of data-mining queries. The same criterion was used to select 90 frog ORs and 12 chicken OR sequences for a fifth round of TBLASTN search. Because the last two rounds did not discover additional ORs, the search was discontinued.

TBLASTN search was conducting setting the parameter -b to 1,000.

All hits that were longer then 30 amino acids and showing at least 30% identity were extracted from the genome and expanded to contain 2,000 bp. These were then translated into proteins and aligned, by CLUSTAL [52], to a multiple alignment of human and mouse ORs [53]. Those that were found to contain the seven-transmembrane domains, and one-third of the amino-terminus and carboxyl-terminus typical lengths were considered automatically as intact ORs; otherwise they were translated via FASTY. The typical OR amino- and carboxyl-termini lengths are based on human and mouse OR repertoires.

To classify an opossum sequence as an OR we required a sequence identity of at least 40% over at least 100 amino acids to any tetrapod OR. Sequences that shared more than 30% sequence identity but less than 40% were searched by BLAST against GPCRDB [54] and all known OR sequences. The score of the best hit from each search was collected. The decision (OR or non-OR) was based on the highest score. Classification into OR families and subfamilies was performed as explained elsewhere [3,13].

### OR genes in alignment chains

An automatic tool (GENETALIGN) was designed to mine and present gene-related information from the UCSC alignment nets [50]. It accepts a list of gene names and coordinates in two aligned species and generates pairs of aligned genes. The 12 alignment nets, which correspond to all available pairwise comparisons among the five mammalian genomes analyzed here (Table 2), were downloaded from the UCSC web page [55]. Pairwise sequence alignment files in AXT format [56] were scanned for alignment blocks whose genomic coordinates overlap with any annotated OR coding sequence in the reference species, and the exact corresponding segments were extracted. Segments shorter than 100 bp in the reference species were filtered out, as were alignments to segments longer than 1,500 bp in the compared species, which are much longer than the typical OR coding sequence. Two alignments separated by no more than 500 bp in both species were joined by adding the required gaps. As a result, a list was constructed using GENETALIGN; for each gene in the reference species, this tool specifies the genomic segments to which the gene is aligned, together with the alignment length and percentage DNA sequence identity. The coordinates of the aligned sequences in the target species were compared with the OR coding sequences annotation of this species. Alignments to genomic segments that were not annotated as ORs, were split between two separated genomic locations, or were located in undefined chromosomal locations were discarded.

To associate genes with alignment chains, alignment annotation files in NET format [56] were scanned for chains that overlap with coordinates of ORs. Only those with an overlap of at least 100 bp were selected. Then, genes associated with a single chain were selected, and the chain number, length, and type were added to the alignment description from the previous step. This procedure was performed also for the chicken versus human alignment net [55].

All analysis procedures were performed on whole-genome alignments.

### Definition of syntenic orthologs

Syntenic orthologs are defined as a pair of ORs from two different species located within the same alignment chain, which is at least 100 kb, and sharing a minimum of 72% DNA sequence identity over the OR coding region. The criterion of 100 kb for minimum chain length was selected to provide a global conservation of genomic neighborhood and usually represents previously defined synteny blocks [50]. The identity value corresponds to half of a standard deviation below the mean sequence identity of all eutherian aligned pairs (78%). Such a subset was defined for every pair of genomes that was analyzed. For the chicken-human comparison, the cutoff of chain length was lowered to 50 kb, and no sequence similarity cutoff was used beyond the maximal expectation value embedded in the alignment chain definition.

### CLIC generation

CLICs are defined over a graph of OR genes (nodes), connected by two types of edges. One type connects pairs of syntenic orthologs, the other type represents immediate neighborhood relations within an OR gene cluster. A CLIC is a connected component of this graph (all the genes connected to each other either directly or via other nodes in the group). Therefore, all genes from one genomic cluster belong to the same component, and all of their orthologs, together with their complete clusters, are also included in this component.

An algorithm to divide a graph into its connected components was constructed using the clustering functions in MATLAB statistics toolbox. It was then applied to the set of 5,969 OR genes from all species. The sequence identity parameter for defining syntenic orthology, as well as the intergenic distance parameter for defining genomic clusters, were aimed to minimize the inclusion of clusters from different chromosomes of the same species in one CLIC.

For each multispecies CLIC, the mean cluster size was calculated, the clusters whose size diverged more than one standard deviation from the mean were excluded from the following calculation, and the recalculated mean served as the estimated consensus cluster size. This was performed to eliminate the effect of species-specific expansion or deletion on the estimated ancestral cluster size.

## Additional data files

The following additional data are available with the online version of this paper. Additional data file 1 is a table listing the complete collection of the 251 CLICs defined in the study, each with the number of genes from each species, and the corresponding cluster names. Additional data file 2 is a table of genomic coordinates of non-OR genes shown in Figure 3. Additional data file3 is a graph of the number of clusters as a function of the maximal intergenic distance parameter. Additional data file 4 is a figure demonstrating the reconstruction of two ancestral clusters. Additional data file 5 is a figure demonstrating conservation of ORs and their genomic regions in the chicken genome. Additional data file6 is a graph describing the length of chicken-human alignment chains containing ORs. Additional data file7 is a graph of the distributions of mutual sequence identity of aligned OR gene pairs identified by GENETALIGN. Additional data file 8 is a table of the genomic coordinates and cluster assignment for all OR genes used in this study. Additional data file 9 contains DNA sequences in FASTA format of the opossum ORs used in this study with their genomic coordinates in the Opossum October 2004 assembly (monDom1). Additional data file 10 contains protein sequences in FASTA format of the opossum ORs used in this study with their genomic coordinates in the Opossum October 2004 assembly (monDom1). Additional data file 11 contains DNA sequences in FASTA format of the dog ORs used in this study with their genomic coordinates in the Dog July 2004 assembly. Additional data file 12 contains protein sequences in FASTA format of the dog ORs used in this study with their genomic coordinates in the Dog July 2004 assembly.

## Supplementary Material

Additional data file 1 Shown is a table listing the complete collection of the 251 CLICs defined in the study, each with the number of genes from each species, and the corresponding cluster names. Click here for file

Additional data file 2 Shown is a table of genomic coordinates of non-OR genes shown in Figure 3. Click here for file

Additional data file 3 The number of OR clusters with at least two genes decreases as we increase the maximal intergenic distance allowed between consecutive genes in the same cluster. This dependency is shown for different species. Click here for file

Additional data file 4 A suggested ancestral configuration is shown at the top row. Genes are presented as parallelograms colored by subfamily affiliation. Inferred chromosomal rearrangements relative to the ancestor are specified for each species: circle = insertion, X = deletion, arrow = inversion, broken line with number of genes = tandem duplication. Click here for file

Additional data file 5 The figure shows schematic alignment of human-chicken genomic regions. Genes shared by human and chicken are shown as filled triangles, and others as empty triangles. Blue = ORs, red = β-globins, purple = taste receptors, green = ephrin receptors. Cluster names are as in Table 3. **(a) **Partial conservation of a human class I OR cluster containing β-globin genes (HBB@). Only a sample of the 103 human ORs is shown. The filled triangles were arbitrarily chosen to demonstrate that the 12 chicken ORs were aligned to 12 different locations in this human cluster. **(b) **Conservation of one human OR gene (OR10AC1P) from CLIC #16 in chicken alongside with partial conservation of syntenic bitter taste (TAS2R39, TAS2R40, TAS2R62P, TAS2R60, TAS2R41) and ephrin receptors (EPHB6, EPHA1). Click here for file

Additional data file 6 For each chicken OR that appears in the chicken human-alignment net, including those that were not assigned a genomic location, the corresponding chain length was recorded. These are sorted from the longest (667026 bp) to the shortest (130 bp), with about 50% longer than 1000 bp. Click here for file

Additional data file 7 The human versus mouse (red) comparison has provided 651 gene pairs with a mean identity value of 75%, compared with 63% for the 693 human-opossum pairs (blue). Click here for file

Additional data file 8 Shown is a table of the genomic coordinates and cluster assignment for all OR genes used in this study. Click here for file

Additional data file 9 Shown are DNA sequences in FASTA format of the opossum ORs used in this study with their genomic coordinates in the Opossum October 2004 assembly (monDom1). Click here for file

Additional data file 10 Shown are protein sequences in FASTA format of the opossum ORs used in this study with their genomic coordinates in the Opossum October 2004 assembly (monDom1). Click here for file

Additional data file 11 Shown are DNA sequences in FASTA format of the dog ORs used in this study with their genomic coordinates in the Dog July 2004 assembly. Click here for file

Additional data file 12 Shown are protein sequences in FASTA format of the dog ORs used in this study with their genomic coordinates in the Dog July 2004 assembly. Click here for file

## Figures and Tables

**Table 1:** A comprehensive collection of OR genes in complete mammalian genomes

Organism	Species name	Genome assembly^a^	Number of OR genes^b^	Number of genomic clusterswith more than one gene	Number of singleton clusters(a single gene)
Human	*Homo sapiens*	hg17	851 (765)	50	30
Dog	*Canis familiaris*	canFam1	922 (804)	45	14
Mouse	*Mus musculus*	mm6	1,296 (1,228)	43	20
Rat	*Rattus norvegicus*	rn3	1,758 (1,654)	53	33
Opossum	*Monodelphis domestica*	monDom1	1,518 (1,518)	92	71
Chicken	*Gallus gallus*	galGal2	554 (45)	7	4

^a^Formal release name as appears in UCSC genome browser [56]. ^b^In parentheses: the number of genes used in this study after discarding genes that are mapped to 'chrUn' or 'random', and human genes from subfamily OR7E. OR, olfactory receptor; UCSC, University of California at Santa Cruz.

**Table 2:** Summary of UCSC pairwise alignments of OR genes

Pair of genomes compared^a^	Total reference OR genes	ORs aligned in the net	ORs aligned to another OR^b^	ORs aligned to a 'syntenic ortholog'^c^	Number of chains containing 'syntenic orthologs'^d^	Correlation between sequence similarity and chain length^e^
Human versus mouse	765	760	651	379 (50%)	33	0.31
Human versus rat	765	763	671	307 (40%)	28	0.21
Human versus dog	765	764	611	391 (51%)	31	0.22
Human versus opossum	765	760	693	109 (14%)	25	0.2
Mouse versus human	1,228	1,222	1,055	376 (31%)	36	0.44
Mouse versus rat	1,228	1,226	1,095	911 (74%)	26	0.43
Mouse versus dog	1,228	1,224	998	395 (32%)	38	0.54
Mouse versus opossum	1,228	1,226	1,119	147 (12%)	30	0.4
Rat versus human	1,654	1,650	1,583	313 (19%)	29	0.22
Rat versus mouse	1,654	1,645	1,400	964 (58%)	32	0.49
Dog versus human	804	804	751	374 (47%)	26	0.26
Dog versus mouse	804	803	683	384 (48%)	36	0.42

^a^Out of 20 possible comparisons between five species, only 12 are available at the UCSC alignment net [56]. A pairwise comparison is directed from a reference genome to a target genome, and is thus not symmetric. ^b^We filtered out alignments between an OR to a genomic segment that was mapped to 'chrUn' or 'random' (approximately 1% of all alignment pairs), was split between two separated genomic locations (approximately 7%), or did not overlap with any annotated OR from the collection described in Table 1 (approximately 3.5%). However, the overlooked segments may contain a genuine OR coding frame, and thus the counts are probably an underestimate for the ORs that have an orthologous counterpart. ^c^The number of alignments that satisfy the criteria of syntenic orthology. The fraction out of the total number of reference genes is given in parentheses. ^d^The total number of alignment chains that together contain all pairs of syntenic orthologs. Usually, each chain contains many such pairs and as such represents a unit of conservation. ^e^Correlation coefficient between the two properties used for defining syntenic orthology: length of the alignment chain from which the aligned gene pair is derived, and the percentage mutual DNA identity between the genes of this pair. Genes with higher identity tend to be in longer chains. OR, olfactory receptor; UCSC, University of California at Santa Cruz.

**Table 3:** Multi-species CLICs of the OR repertoire

CLIC number^a^	Human		Mouse		Rat		Dog		Opossum		Consensus size
	Clusters^b^	Genes (*n*)	Clusters^b^	Genes (*n*)	Clusters^b^	Genes (*n*)	Clusters^b^	Genes (*n*)	Clusters^b^	Genes (*n*)	
1	-	0	chr4@117.8	15	chr5@139.3	15	chr15@3.3	4	s13629@2.4	6	12
4	chr1@155.4 chr1@156.2	31	chr1@173 chr1@174.2	21	chr13@89.5 chr13@90.2	28	chr38@19.8	27	s15142@1.8 s16926@0.2 s19280@0.6	31	29
5	chr1@244.6	56	chr11@58.4 chr11@59.3 chr16@18.2 chr7@80.4	49	chr10@44.6 chr10@45.9 chr11@83.7 chr1@142.7	79	chr14@4.6 chr16@4.4 chr8@3.6	53	s13645@0.9	18	53
9	chr3@99.5	18	chr16@58.1	28	chr11@42.2	36	chr33@8.3	11	s12721@4.5	12	17
11	chr5@180.1 chr5@180.6	5	chr11@49.1	16	chr10@34.3 chr10@34.9	19	-	0	s16810@0.6	5	9
12	chr6@28.1 chr6@28.5 chr6@29.4	34	chr13@20.9 chr17@35.5	63	chr17@50.6 chr17@51.3 chr20@0.8	85	chr35@28.1 chr35@29.2	10	s14804@0.5	27	41
16	chr7@142.7 chr7@143.3	21	chr6@43	23	chr4@70.9	20	chr16@11.7	19	s12761@1.3	24	21
17	chr9@35.9	7	chr4@43.7	6	chr5@60.2	8	chr11@53.8	8	-	0	8
19	chr9@104.5	12	chr4@52.8	5	chr5@70.2	11	chr11@61.9	12	s18607@0.4	22	12
21	chr9@122.5	15	chr2@36.7	34	chr3@16	39	chr9@52.6	8	s15087@1.4	18	22
23	chr11@5.2	103	chr7@97.5 chr7@99.1	146	chr1@161.7	149	chr21@30.7	111	s15168@3.2 s16805@1.4	149	139
24	chr11@6.8	8	chr7@100.9	24	chr1@164.2	31	chr21@32.6	24	-	0	26
25	chr11@7.8	8	chr7@102.3	41	chr1@166	47	chr21@33.7	9	-	0	19
26	chr11@48.4 chr11@50 chr11@51.3 chr11@55.7	146	chr2@87.6	251	chr3@71.6	300	chr18@50.7	144	s13644@1 s18549@1.3 s19209@1.4	281	266
27	chr11@57.7 chr11@59.1	42	chr19@12.1	76	chr1@215.2 chr1@216.5	66	chr18@47.9 chr18@48.7	40	s12795@1.2 s12795@2.8 s12795@3.4	111	56
29	chr11@123.6	44	chr9@38.9	112	chr8@39.3 chr8@41 chr8@42.7	139	chr5@13.2	44	s18579@6.8 s18622@0.4	77	69
30	chr12@47.1	8	chr15@98.4	7	chr7@137.2	8	chr27@9.2	22	-	0	8
31	chr12@54.1	28	chr10@129.3	58	chr7@5.4	194	chr10@19.4 chr10@3.1 chr27@3.2 chr3@34.2	49	s12526@0.2 s15221@0.8	82	54
32	chr14@19.5 chr15@100.2 chr15@19.8	46	chr14@45.4 chr2@111.3	64	chr15@26.3 chr3@97.3	68	chr15@20.4 chr30@3.3	39	s11704@0.4 s19262@7	74	59
35	chr14@21.2	5	chr14@47.5	6	chr15@27.9	7	chr15@21.6	2	s19262@4.7	8	6
39	chr17@3.1	16	chr11@73.6	43	chr10@61	49	chr9@39.8	15	-	0	25
42	chr19@9.2	10	chr9@19.4	43	chr8@16.2 chr8@18.1	74	chr20@54.4	20	-	0	24
45	chr19@14.9	14	chr10@78.9	8	chr7@12.4	16	chr20@50.3	41	s11688@0.2	11	12
46	chr19@15.9	6	chr8@71.2	3	chr16@18.2	1	chr20@49.3	16	s11661@2.3	16	5
48	chrX@130.3	9	chrX@44.5 chrX@44.9	3	chrX@136.4 chrX@137.1	5	chrX@105.6	3	s11989@0.2	9	4

^a^The CLICs are ordered according to genomic order in the human genome. For CLICs that do not contain human clusters, the human location that is syntenic to the region of the mouse OR cluster was considered (according to UCSC mm6 versus hg17 alignment net [56]). Only multi-species CLICs with at least five human genes are shown, in addition to CLIC #1, which is discussed in the text. The complete list of 251 CLICs appear in Additional data file 1. ^b^Cluster names indicate the chromosome (or the scaffold for the opossum genome) followed by the genomic coordinates in megabases of the middle of the cluster. CLIC, clusters in conservation; OR, olfactory receptor; UCSC, University of California at Santa Cruz.
