# Supplementary figures and images for: Ancient genomic architecture for mammalian olfactory receptor clusters
Source: Genome Biol. 2006 Oct 1;7(10):R88. doi: 10.1186/gb-2006-7-10-r88 (PMC1794568; doi:10.1186/gb-2006-7-10-r88)

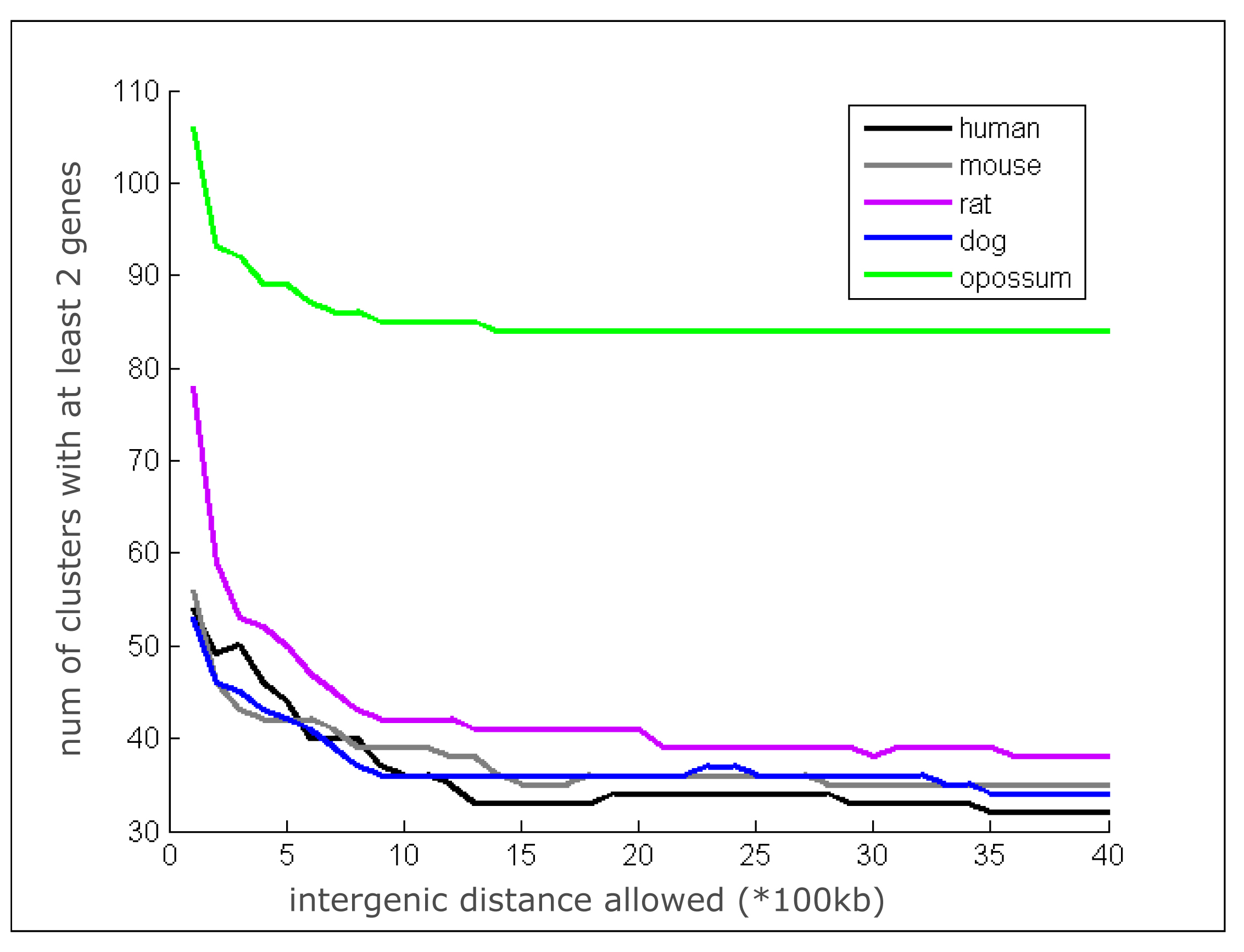

Supplement: Additional data file 3 — The number of OR clusters with at least two genes decreases as we increase the maximal intergenic distance allowed between consecutive genes in the same cluster. This dependency is shown for different species. [file gb-2006-7-10-r88-S3.jpeg]

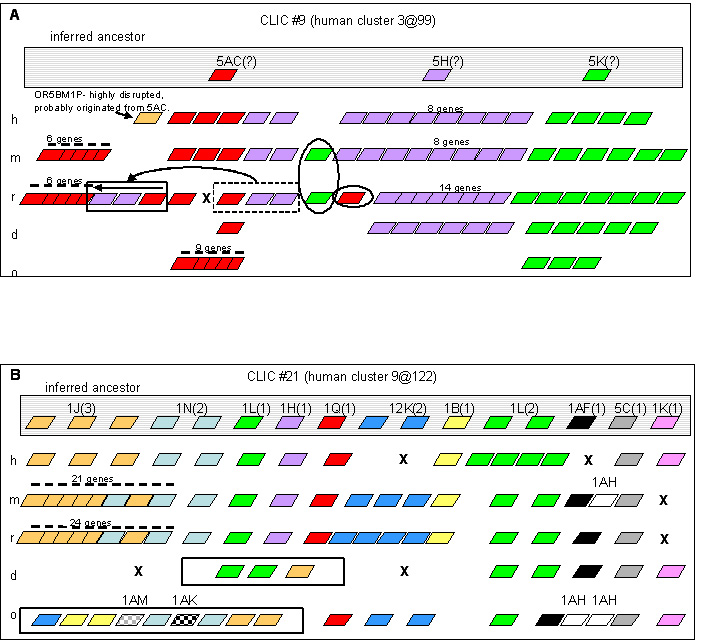

Supplement: Additional data file 4 — A suggested ancestral configuration is shown at the top row. Genes are presented as parallelograms colored by subfamily affiliation. Inferred chromosomal rearrangements relative to the ancestor are specified for each species: circle = insertion, X = deletion, arrow = inversion, broken line with number of genes = tandem duplication. [file gb-2006-7-10-r88-S4.jpeg]

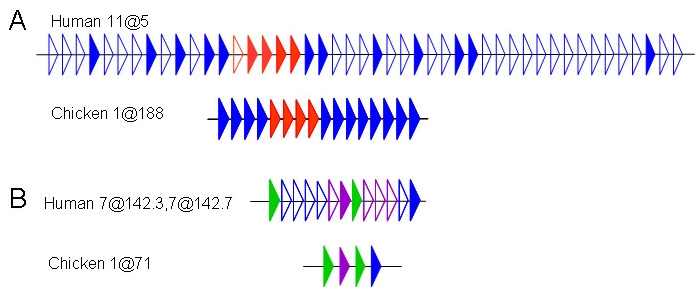

Supplement: Additional data file 5 — The figure shows schematic alignment of human-chicken genomic regions. Genes shared by human and chicken are shown as filled triangles, and others as empty triangles. Blue = ORs, red = β-globins, purple = taste receptors, green = ephrin receptors. Cluster names are as in Table 3. (a) Partial conservation of a human class I OR cluster containing β-globin genes (HBB@). Only a sample of the 103 human ORs is shown. The filled triangles were arbitrarily chosen to demonstrate that the 12 chicken ORs were aligned to 12 different locations in this human cluster. (b) Conservation of one human OR gene (OR10AC1P) from CLIC #16 in chicken alongside with partial conservation of syntenic bitter taste (TAS2R39, TAS2R40, TAS2R62P, TAS2R60, TAS2R41) and ephrin receptors (EPHB6, EPHA1). [file gb-2006-7-10-r88-S5.jpeg]

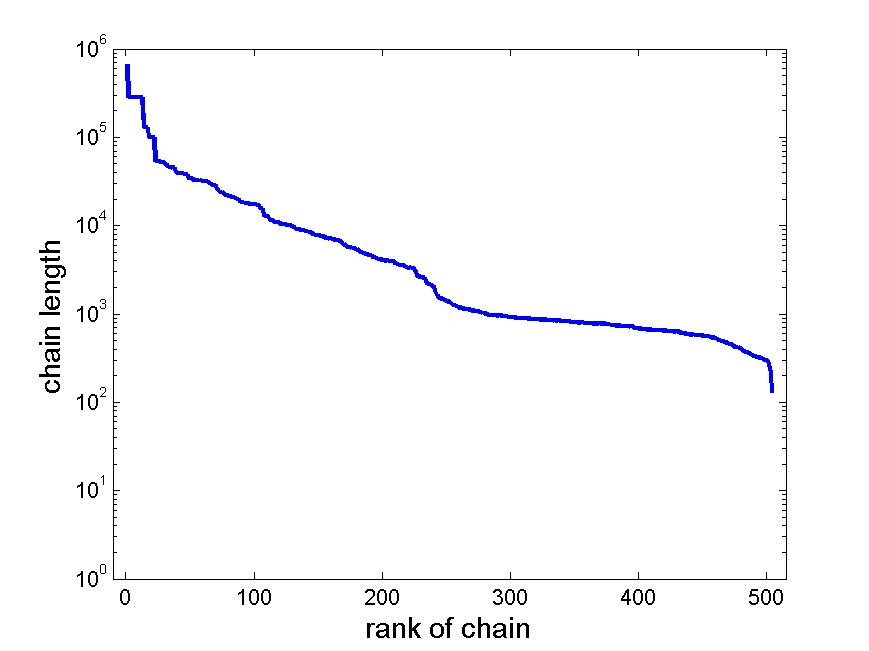

Supplement: Additional data file 6 — For each chicken OR that appears in the chicken human-alignment net, including those that were not assigned a genomic location, the corresponding chain length was recorded. These are sorted from the longest (667026 bp) to the shortest (130 bp), with about 50% longer than 1000 bp. [file gb-2006-7-10-r88-S6.jpeg]

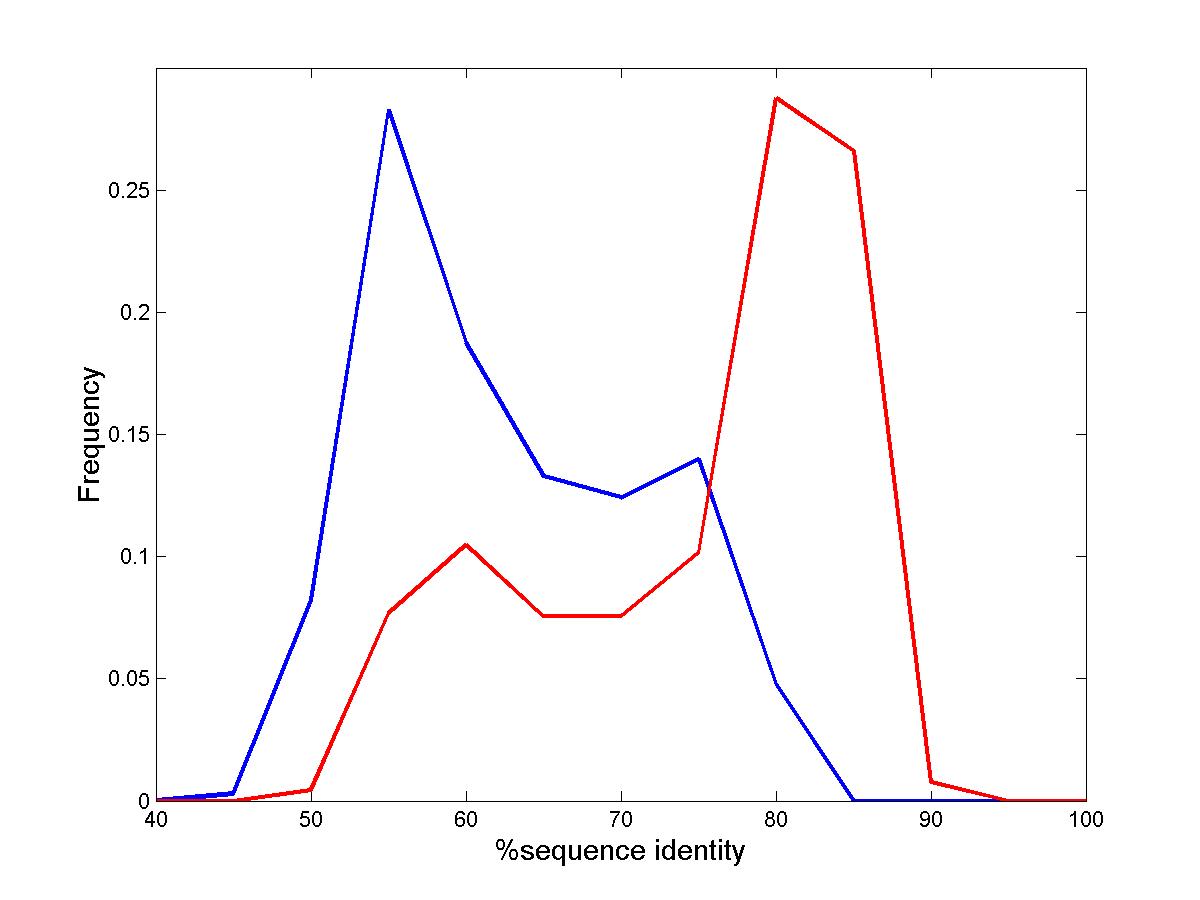

Supplement: Additional data file 7 — The human versus mouse (red) comparison has provided 651 gene pairs with a mean identity value of 75%, compared with 63% for the 693 human-opossum pairs (blue). [file gb-2006-7-10-r88-S7.jpeg]
